# Anti-Hypertensive Herbs and Their Mechanisms of Action: Part II

**DOI:** 10.3389/fphar.2016.00050

**Published:** 2016-03-08

**Authors:** M. Akhtar Anwar, Sara S. Al Disi, Ali H. Eid

**Affiliations:** ^1^Department of Biological and Environmental Sciences, Qatar UniversityDoha, Qatar; ^2^Department of Pharmacology and Toxicology, Faculty of Medicine, American University of BeirutBeirut, Lebanon

**Keywords:** herbal medicine, hypertension, endothelial/vascular smooth muscle cells, oxidative stress, inflammation, nitric oxide, epigenetics

## Abstract

Traditional medicine has a history extending back to thousands of years, and during the intervening time, man has identified the healing properties of a very broad range of plants. Globally, the use of herbal therapies to treat and manage cardiovascular disease (CVD) is on the rise. This is the second part of our comprehensive review where we discuss the mechanisms of plants and herbs used for the treatment and management of high blood pressure. Similar to the first part, PubMed and ScienceDirect databases were utilized, and the following keywords and phrases were used as inclusion criteria: hypertension, high blood pressure, herbal medicine, complementary and alternative medicine, endothelial cells, nitric oxide (NO), vascular smooth muscle cell (VSMC) proliferation, hydrogen sulfide, nuclear factor kappa-B (NF-κB), oxidative stress, and epigenetics/epigenomics. Each of the aforementioned keywords was co-joined with plant or herb in question, and where possible with its constituent molecule(s). This part deals in particular with plants that are used, albeit less frequently, for the treatment and management of hypertension. We then discuss the interplay between herbs/prescription drugs and herbs/epigenetics in the context of this disease. The review then concludes with a recommendation for more rigorous, well-developed clinical trials to concretely determine the beneficial impact of herbs and plants on hypertension and a disease-free living.

## Herbs and spices less commonly used for treatment of hypertension

In the first part of this review (Al Disi et al., 2015), we discussed the molecular and cellular mechanisms involved in the pathogenesis of hypertension. We followed that by an in-depth discussion of the different herbs/spices that are most commonly used for the management and treatment of hypertension (Al Disi et al., 2015). However, there are many other herbs/spices that appear to have significant effects in favorably modulating high blood pressure. Yet, these herbs are not very commonly used, due to several reasons, such as restriction of their growth habitat or use by few or disparate nations and tribal groups. Moreover, limited knowledge in terms of research evidence and wisdom restricted to certain cultures, tribal groups, or nations of small effective population size are potential contributors to this infrequent use and scarcity of research-based evidence.

Here, in this second part of our review, we discuss the effects and mechanisms of action for herbs/spices that are less commonly consumed. We present these plants in an alphabetical order and then summarize their effects and mechanisms of action. Attention in the tables is given to plants that have antioxidant (Table 1), vasorelaxant (Table 2), anti-inflammatory (Table 3), anti-proliferative (Table 4), or diuretic effects (Table 5). Table 6 then summarizes the findings for the plants/herbs which have been studied in clinical trials.

**Table 1:** **Less commonly used antihypertensive plants with antioxidant activity**.

**Herb**	**Effect/Mechanism**	**Concentration/Dose**	**Experimental setting/Model**	**References**
*Agelanthus dodoneifolius*	Scavenges ROS	0.125 mg/ml	DPPH enzymatic assay	Builders et al., 2012
*Alpinia zerumbet*	Reduces oxLDL	0.1 mg/L	Human umbilical vein endothelial cells	Shen et al., 2012
*Apocynum venetum*	Scavenges ROS	10 μg/ml	Rat isolated aortic rings	Lau et al., 2012
*Arctium lappa*	Scavenges ROS	4.79 μg/ml	DPPH enzymatic assay	Predes et al., 2011
*Cnidium monnieri*	Increases antioxidants	20 mg/kg	Renovascular hypertensive rats	Zhou et al., 2012
*Cnidium officinale*	Scavenges ROS	0.32–200 μg/ml	DPPH enzymatic assay	Jeong et al., 2009
*Desmodium gangeticum*	Reduces ROS	50–200 μg/ml	Isoproterenol-treated cardiomyocytes	Sankar et al., 2013
*Elettaria cardamomum*	Increases antioxidants	3 g/day	Stage 1 hypertensive patients	Verma et al., 2009
*Embelia ribes*	Increases antioxidants	100 mg/kg	Isoproterenol-treated rats	Bhandari et al., 2008a
		50 mg/kg	High fat-fed rats	Chaudhari et al., 2012
*Ferula gummosa*	Increases antioxidants	90 mg/kg	SHR	Gholitabar and Roshan, 2013
*Gastrodia elata Blume*	Decreases LDL cholesterol	6 mg/kg/day	High fat-fed SHR	Lee O. H. et al., 2012
*Kalanchoe pinnata*	Increases antioxidants	25–100 mg/kg/day	High salt-loaded rats	Bopda et al., 2014
*Lepidium sativum*	Scavenges ROS	500 μg (of the lyophilized extract in a tube)	DPPH enzymatic assay	Kaur et al., 2013
*Melothria maderaspatana*	Increases antioxidants	50, 100, and 200 mg/kg	DOCA-salt hypertensive rats	Veeramani et al., 2011
*Ocimum basilicum*	Scavenges ROS	IC_50_ range: 8.17–24.91 μg/ml (for different solvent-extractions)	DPPH enzymatic assay	Kaurinovic et al., 2011
*Phyllanthus amarus*	Increases antioxidants	200 mg/kg	Male Albino Wistar rats	Karuna et al., 2009
	Scavenges oxidants	0–1 mg/ml (varies for each assay)	DPPH enzymatic assay (and other oxidant scavenging assays)	Maity et al., 2013
*Picrasma quassiodes*	Regulates SOD and NO	100 and 200 mg/kg	SHR	Zhao et al., 2013
*Pueraria lobata*	Scavenges oxidants	IC_50_ range: 7.90 to >200 μg/ml (for different solvent-extractions)	DPPH enzymatic assay (and other *in vitro* assays)	Jin et al., 2012
*Radix Angelicae*	Increases antioxidants	6.25–25 mg/kg/day	2K-1C rats	Cao et al., 2013
	Decreases peroxidants			
*Raphanus sativus*	Increases antioxidants	30 and 90 mg/kg	SHR	Chung et al., 2012
	Scavenges oxidants	IC_50_ range: 23–200 μg/ml (for different parameters using different solvent-extractions)	DPPH enzymatic assay	Beevi et al., 2010
*Rhus coriaria*	Scavenges oxidants	IC_50_ range: 0.71 to >12 μg/ml (for different fractions)	DPPH enzymatic assay	Kosar et al., 2007
*Sambucus ebulus L*.	Increases antioxidants	200 ml/day	Healthy humans	Ivanova et al., 2014
*Sesamum indicum*	Scavenges ROS	25–1000 mg/ml	DPPH enzymatic assay	Visavadiya et al., 2009
	Inhibits LDL peroxidation		Mitochondrial fraction and human serum	
*Stephania tetrandra*	Reduces iNOS and COX-2 expression	100 μM of its active compound, tetrandrine	Human monocytic cells	Wu and Ng, 2007
	Reduces production of oxidants	25–50 μM of its active compound, tetrandrine	LPS-stimulated microglia	Xue et al., 2008
*Suaeda asparagoides*	Scavenges ROS	IC_50_ range: 9–42 μg/ml (for different solvent-extractions)	DPPH enzymatic assay	Park et al., 2007
*Tribulus terrestris*	Reduces H_2_O_2_	0.240 mg/ml of the plant's total saponins	Primary VSMCs from aorta of newborn calves	Li et al., 2013
*Tropaeolum majus L*	Reduces ACE	300 mg/kg ethanolic extract, 200 mg/kg purified fraction, 10 mg/kg isoquercitrin	SHRs	Gasparotto Junior et al., 2012
	Reduces ROS	10 mg/kg		
*Viscum articulatum Burm*	Inhibits cardiac lipid peroxidation	60 mg/kg/day	Glucocorticoid-treated rats	Bachhav et al., 2011
	Increases NO			
	Scavenges oxidants	ED_50_ range: 34.1–63.3 μg/ml (for different compounds)	DPPH enzymatic assay	Kuo et al., 2010

**Table 2:** **Less commonly used antihypertensive plants with vasorelaxant activity**.

**Herb**	**Effect/Mechanism**	**Concentration/Dose**	**Model**	**References**
*Agelanthus dodoneifolius*	Not clear	0.001–3 mg/ml	Rat isolated aorta	Ouedraogo et al., 2011
*Acorus calamus*	Ca^2+^ regulation	0.01–1 mg/ml	High K^+^-treated rabbit aorta	Shah and Gilani, 2009
*Allium cepa*	Not clear	0.0625–2 mg/ml	Rat isolated thoracic aorta	Naseri et al., 2008
	Increases eNOS expression	400 mg/kg/day	Fructose-fed rats	Vazquez-Prieto et al., 2011
*Alpinia zerumbet*	Increases NO	100 and 300 μg/ml	DOCA-salt-induced hypertension in rats	de Moura et al., 2005
	Blocks Ca^2+^ channels	1–20 mg/kg	Wistar rats thoracic aortic rings	da Cunha et al., 2013
*Apocynum venetum*	Increases NO production	10 μg/ml	Rat isolated aortic rings	Lau et al., 2012
*Arctium lappa*	Not clear	100 and 200 mg/kg/day	High fat-fed Sprague-Dawley rat thoracic aorta	Lee Y. J. et al., 2012
*Artemisia verlotorum* Lamotte	Increases NO	10^−5^–10^−3^ g/ml	Rat isolated aorta	Calderone et al., 1999
*Bacopa monnieri*	Ca^2+^ regulation	102–494 μg/ml	Various isolated blood vessels from rat	Kamkaew et al., 2011
	Increases NO			
*Carthamus tinctorius L*	Opens K_ATP_ channels	0.1–3 mg/kg/day	Rat isolated hearts	Nie et al., 2012
*Cassia occidentalis*	Blocks Ca^2+^ influx	3.06 and 3.63 mg/ml	Rat isolated aorta	Ajagbonna et al., 2001
*Cinnamomum zeylanicum*	Opens K_ATP_ channels	5, 10, and 20 mg/kg	Isolated aortic rings	Nyadjeu et al., 2011
	Increases NO	300 mg/kg/day	Isolated aortic rings	Nyadjeu et al., 2013
*Cirsium japonicum*	Increases NO bioavailability	0.1–1 mg/ml	Rat isolated thoracic aorta	Kim et al., 2008
*Coleus forskohlii*	Relaxes vessels	ED50: Rat aorta: 37 nM Bovine coronary: 51 nM; Canine coronary: 0.3 μM	rat aorta, bovine coronary artery, canine coronary artery	Muller and Baer, 1983
	Lowers blood pressure	Up to 5 μg/ml	Feline, canine, SHR and renal hypertensive rats	Lindner et al., 1978
	Reduces MAP; mechanism not elucidated but likely via cAMP	0.1–1 mg/ml; *i.v*.	Feline	Dubey et al., 1981
		10 mg/kg, p.o.	Normotensive rats and SHR	Dubey et al., 1981
	Decreases SBP, DBP, and MAP; mechanism not elucidated but likely via cAMP	0.5–3 μg/kg/min infusions of forskolin	Human patients with cardiomyopathy	Baumann et al., 1990; Schlepper et al., 1989
*Crotalaria sessiliflora*	Promotes extracellular Ca^2+^ influx and release of Ca^2+^ from intracellular stores	0.5–5 mg/ml	Rat thoracic aortic rings	Koh et al., 2007
	Increases NO			
*Cynanchum wilfordii*	Increases Akt, NO and eNOS	100 and 200 mg/kg/day	High fat/cholesterol-fed rats	Choi et al., 2012a
	Increases NO and cGMP levels	100 and 200 mg/kg/day	High fat/cholesterol-fed ApoE-deficient mice	Choi et al., 2012b
*Echinodorus grandiflorus*	Increases NO production	300–1000 mg/kg; *i.v*.	SHR	Lessa et al., 2008
		0.1 mg/ml	Rabbit aortic rings	Tibiriçá et al., 2007
*Elettaria cardamomum*	Blocks Ca^2+^ channels	2.94 mg/ml	Rat isolated aorta	Gilani et al., 2008
*Euphorbia humifusa*	Ca^2+^ regulation	10^−4^–10^−7^ g/ml	Rat aortic rings	Wang et al., 2013
	Increases NO			
*Gentiana floribunda*	Blocks Ca^2+^ channels	0.3–1 mg/ml	Rat aorta	Khan et al., 2012
*Geum japonicum*	Increases NO	30 μg/ml	Rat aortic rings	Xie et al., 2007
*Gynura procumbens*	Inhibits Ca^2+^ channels	10^−5^–10^−3^ g/ml	Rat aortic rings	Hoe et al., 2011
	Increases NO	500 mg/kg	SHR	Kim et al., 2006
	Opens K_ATP_ channels	0.003 and 0.009 g/ml	Rat isolated thoracic aortic rings	Ng et al., 2013
*Kaempferia parviflora*	Reduces Ca^2+^ influx	10 and 100 μM	Rat isolated aortic rings	Tep-Areenan et al., 2010
	Decreases defibrillation efficacy	12.5–100 mg/kg	Porcine	Weerateerangkul et al., 2013
	Decreases Ca^2+^	75–300 μg/mL	Ventricular myocytes	Weerateerangkul et al., 2012
	Increases NO signaling	1–100 μM	Rat isolated aortic rings	Tep-Areenan et al., 2010
		100 mg/kg	Rat heart	Weerateerangkul et al., 2012
*Lavandula stoechas*	Ca^2+^ antagonists	0.1–1 mg/ml	Rabbit intestines	Gilani et al., 2000
*Marrubium vulgare*	Likely acts via the NO pathways,	80 mg dry extract/kg, once a day, for 5 days.	Rat aorta	El Bardai et al., 2001
	Blocks L-type calcium channels	1–30 μM of marrubenol (a component of *M. vulgare*)	Endothelial denuded aortic rings	El-Bardai et al., 2003
*Melothria maderaspatana*	Decreases endothelin, epinephrine and norepinephrine	60 mg/kg	DOCA-salt-sensitive hypertensive rats	Veeramani et al., 2012
*Microdesmis keayana*	Increases eNOS expression	0.5–50 mg/ml	Rat and guinea-pig aortic strips	Zamble ´ et al., 2006
*Moldenhawera nutans*	Regulates sympathetic tone	1–10 mg/kg	Rat isolated aorta	Lahlou et al., 2007
*Nitraria sibirica Pall*.	Increases NO	0.1–10 g/l	Rat isolated thoracic aortic rings	Senejoux et al., 2012
*Ocimum basilicum*	Ameliorates lipidemia-induced endothelial dysfunction	0.5 g/kg body weight	Rat isolated aortic rings	Amrani et al., 2009
	Decreases ET-1 and Ang II levels	100, 200, and 400 mg/kg	Renovascular hypertensive rats	Umar et al., 2010
*Oenothera odorata*	Increases NO	5.16 μg/ml	Rat carotid arterial rings	Kim et al., 2011
*Peganum harmala*	Increases NO	3–30 μM	Rat isolated rat aorta	Shi et al., 2001; Berrougui et al., 2006
*Petasites formosanus*	Antagonizes Ca^2+^	10–100 μM	Rat aortic smooth muscles	Wang et al., 2002
*Peucedanum praeruptorum Dunn*	Increases NO	1–100 μM	Rat isolated aortic rings	Xu et al., 2010
*Piper nigrum*	Antagonizes Ca^2+^	1–20 μM	Rabbit aortic rings	Taqvi et al., 2008
*Piper truncatum*	Increases NO	10.7 μg/ml	Rat aorta	Raimundo et al., 2009
	Regulates histamine receptors			
*Prunella vulgaris*	Increases NO	100 and 200 (mg/kg)	Db/db mice with type 2 diabetes	Hwang et al., 2012
*Pseuderanthemum palatiferum*	Increases NO	81 μg/ml	Rat endothelial-intact aortic ring	Khonsung et al., 2011
*Pueraria lobata*	Opens K_ATP_ channels	1 mg/ml of the extract; 1-10 nM of puerarin, a compound *of P. lobata*	Rat thoracic aorta	Sun et al., 2007; Ng et al., 2011
*Radix Angelicae*	Decreases Ang II levels	6.25–25 mg/kg/day	2K-1C rats	Cao et al., 2013
*Ranunculus japonicus*	Reduces Ang II concentration	0.2 mg/ml (of the plant's glycosides)	2K-1C rats	Liu et al., 2012
*Raphanus sativus*	Increases NO	30 and 90 mg/kg	SHR	Chung et al., 2012
		0.3–3 mg/ml	Rat Isolated aorta	Ghayur and Gilani, 2006
			Guinea-pig atria	
*Rhazya stricta*	Reduces BP and HR; unclear mechanism	0.5–2 mg/animal	Rats	Tanira et al., 2000
*Rhus coriaria*	Activates eNOS	0.3–300 μg/ml	Rabbit isolated aortic rings	Beretta et al., 2009
*Sesamum indicum*	Increases NO	180 mg/ml	Rat Isolated aorta	Suresh Kumar et al., 2008
*Solanum sp*	Increases NO	75 μg/ml	Rat aortic rings	Monteiro et al., 2012
*Sorbus cortex*	Regulates NO and ET-1	100 and 200 mg/kg	Rats with atherogenic diet	Sohn et al., 2005
*Stephania tetrandra*	Blocks Ca^2+^ channels	3–30 mg/kg (*i.v*.) or 10–30 μM of tetrandrine in the pulmonary artery;	Rats	Qian et al., 1983
*Tribulus terrestris*	Increases NO	0.3–15 mg/ml	SHR	Phillips et al., 2006
	Reduces ACE	10 mg/kg	2K-1C rats	Sharifi et al., 2003
*Tridax procumbens*	Not clear	0.15–1.05 mg/ml	Rat isolated aortic rings	Salahdeen and Murtala, 2012
*Tropaeolum majus*	Inhibits ACE	30–300 mg/kg of hydroethanolic extract, or 25–100 mg/kg of semi-purified fraction	ACE activity assay (in serum from normotensive Wistar rats)	Gasparotto Junior et al., 2012
	Increases NO			
*Uncariae ramulus et Uncus*	Increases NO	450 mg/kg/day	SHR	Goto et al., 1999
*Viola odorata*	Increases NO	0.39 and 0.4 mg/ml	Guinea-pig atria and rat aorta	Siddiqi et al., 2012
	Regulates Ca^2+^	0.3–3 mg/ml		
*Viscum articulatum Burm*	Increases NO	60 mg/kg/day	Glucocorticoid-treated rats	Bachhav et al., 2011
*Zizyphi Spinosi*	Increases NO	0.1–100 μM	L-NAME treated rats	Fu et al., 2011

**Table 3:** **Less commonly used antihypertensive plants with anti-inflammatory activity**.

**Herb**	**Effect/Mechanism**	**Concentration/Dose**	**Model**	**References**
*Arctium lappa*	Suppresses VCAM-1 (aortic endothelia)	100 and 200 mg/kg/day	High fat-fed Sprague-Dawley rat thoracic aorta	Lee Y. J. et al., 2012
*Carthamus tinctorius*	Decreases soluble (plasma) VCAM-1	2.1 g/day	Healthy humans	Koyama et al., 2009
*Cirsium japonicum*	Decreases NF-κB expression (mast cells)	0.05–0.4 mg/ml	HMC-1 human mast cells	Kim et al., 2013
*Cuminum cyminum*	Decreases TNF- α and IL-6 (renal tissue)	200 mg/kg	renovascular hypertensive rats	Kalaivani et al., 2013
*Cynanchum wilfordii*	Inhibits VCAM-1 and ET-1 activity (aortic endothelia)	100 and 200 mg/kg/day	High fat/cholesterol-fed ApoE-deficient mice	Choi et al., 2012b
*Gastrodia elata Blume*	Decreases iNOS expression (gastric mucosa)	0.02 mL/g	Stress-induced gastric lesions in mice	An et al., 2007
*Phyllanthus amarus*	Decreases NF-κB, TNF-α, and COX-2 (RAW 264.7 cells)	0–250 μg/ml (aqueous ethanol) or 0–200 μg/ml (hexane) fractions	LPS-treated RAW 264.7 macrophages	Kiemer et al., 2003
*Pueraria lobata*	Decreases expression of iNOS (RAW 264.7 cells)	20 and 100 μM of lupenone or lupeol, respectively	LPS-treated RAW 264.7 macrophages	Jin et al., 2012
*Radix Astragali*	Attenuates IFN-γ and IL-2 (serum of rats)	0.4 g of the plant per 100g of the animal weight	Rat model of autoimmune myocarditis	Zhao et al., 2008
*Raphanus sativus*	Reduces LPS-induced NO, IL-1β, TNF-α, and IFN-γ (RAW 264.7 cells)	100 μg/ml of each sub-fraction	LPS-stimulated RAW264.7macrophages	Kook et al., 2014
*Rheum rhabarbarum*	Reduces LPS-induced NO, IL-6, IL-1β, IL-8, or TNF-α (colon tissue or RAW 264.7 cells)	100 mg/kg rhein (in mice) or 8–40 μM of different anthraquinones (in RAW264.7 cells)	Mice or RAW264.7 cells	Hu et al., 2014; Zhang et al., 2015
*Sophora flavescens Ait*	Reduces expression of IL-6, VCAM-1, and ET-1 (pulmonary artery endothelia)	0.5–2 mg/mL	Monocrotaline-induced pulmonary hypertension in rats	Zhang et al., 2014
*Sorbus cortex*	Regulates NO and ET-1 (aortic endothelia)	100 and 200 mg/kg	Rats with atherogenic diet	Sohn et al., 2005
*Stephania tetrandra*	Inhibits LPS-induced expression of PgE_2_, iNOS, and COX-2 (THP-1 monocytic cell line)	100 μM of tetrandrine (a component of *S. tetrandra*)	THP-1 cells	Wu and Ng, 2007
	Reduces LPS-induced levels of IL1β and TNFα (BV-2 microglia)	0.1–1 μM of tetrandrine (a component of *S. tetrandra*)	BV-2 microglia	Dang et al., 2014

**Table 4:** **Less commonly used antihypertensive plants with antiproliferative activity**.

**Herb**	**Effect/Mechanism**	**Concentration/Dose**	**Model**	**References**
*Angelica sinensis and Ligusticum chuanxiong*	Arrests VSMCs at G0/G1	300 μg/ml	Rat vascular smooth muscles	Hou et al., 2005
*Raphanus sativus*	Arrests VSMCs in G1 phase	20–160 μg/ml	Mouse aortic smooth muscle cells	Suh et al., 2006
*Sophora flavescens Ait*	Inhibits hypoxia or TGF-β-induced proliferation	0.5–0.2 mg/ml	Pulmonary arterial smooth muscle cells	Zhang et al., 2014
*Tribulus terrestris*	Inhibits Ang II-induced proliferation	0.240 mg/ml of the plant's total saponins	Primary VSMCs from aorta of newborn calves	Li et al., 2013

**Table 5:** **Less commonly used antihypertensive plants with diuretic activity**.

**Herb**	**Effect/Mechanism**	**Concentration/Dose**	**Model**	**References**
*Elettaria cardamomum*	Increases urine output and enhances Na^+^ and K^+^ excretion	1, 3, and 10 mg/kg	Anesthetized rats	Gilani et al., 2008
*Lepidium latfolium*	Increases urine output and electrolyte excretion	50–100 mg/kg	Rats	Navarro et al., 1994
*Lepidium sativum*	Increases electrolyte excretion	20 mg/kg	SHR	Maghrani et al., 2005
*Phyllanthus amarus*	Increases urine volume and Na^+^ levels in serum (humans) and decreases SBP and DBP (in man)	80 mg/kg (in rabbits)	Mild hypertensive patients and rabbits	Srividya and Periwal, 1995; Amaechina and Omogbai, 2007
*Tropaeolum majus L*	Reduces aldosterone	300 mg/kg ethanolic extract, 200 mg/kg purified fraction, 10 mg/kg isoquercitrin	SHR	Gasparotto Junior et al., 2012
	Downregulates renal Na^+^/K^+^ pump			
	Increases urine volume			
*Viscum articulatum Burm*	Increases urine volume	200 mg/kg/day	L-NAME-treated rats	Bachhav et al., 2012
	Increases urine volume, electrolyte excretion and glomerular filtration rate	100, 200, and 400 mg/kg	Male Wistar rats	Jadhav et al., 2010

**Table 6:** **Less commonly used antihypertensive plants used in clinical trials**.

**Herb**	**Design**	**Population Size**	**Condition**	**Dose**	**Duration**	**Effect**	**Magnitude of change**	**References**
*Carthamus tinctorius L*	Double-blind, placebo-controlled	92	Mild hypertension	70 mg/day ethanolic safflower extract	12 weeks	No significant SBP or DBP decrease	0.8/1 mmHg	Suzuki et al., 2010
*Elettaria cardamomum*	Placebo-controlled trial	20	Stage 1 hypertension	3 g cardamom powder (1.5 g capsule, twice a day)	12 weeks	SBP and DBP decrease	19/12 mmHg	Verma et al., 2009
*Melothria maderaspatana*	Controlled trial	50	Mild hypertension	4% tea leaf powder	45 days	SBP and DBP decrease	23.8/15.5 mmHg	Raja et al., 2007

### *Acorus calamus* (sweet flag or calamus)

Different solvent extracts of *Acorus calamus* have been reported to decrease mean arterial pressure (MAP) in normotensive rats (Shah and Gilani, 2009; Table 2). This plant can cause vasoconstrictive or vasodilatory activities on baseline and high K^+^-induced contractions in rabbit aorta, suggesting that it may regulate vascular tone (Shah and Gilani, 2009). Its effects seem to be mediated through a Ca^2+^-dependent mechanism (Shah and Gilani, 2009).

### *Agelanthus dodoneifolius* (mistletoe)

Ethanolic extracts of *Agelanthus dodoneifolius* (mistletoe) (0.001–3 mg/ml) showed dose-dependent relaxing effects on rat isolated aortic rings (Ouedraogo et al., 2011). In addition, 0.01–10 mg/ml of the same extract decreased systolic blood pressure (SBP) and diastolic blood pressure (DBP) in normotensive rats (Ouedraogo et al., 2011; Table 2). The active component with the most potent activity was identified as dihydropyranone dodoneine (Ouedraogo et al., 2011). While this plant appears to exhibit antioxidant activity (Builders et al., 2012; Table 1), further investigations are warranted to decipher the mechanism relevant to hypotensive effect. A potential mechanism was recently proposed, wherein dodoneine was shown to induce vasorelaxation by inhibiting carbonic anhydrase and activating calcium-gated potassium (K_Ca_) channels (Carre et al., 2015) as well as precipitating a negative inotropic effect on the rat heart (Carré et al., 2014).

### *Allium cepa* (onion)

*Allium cepa*, or onion, has been reported to reduce BP in fructose-fed (Naseri et al., 2008; Table 2) and anesthetized normotensive rats (Brankovic et al., 2011). Aqueous extracts of onion (400 mg/kg/day) increase expression of endothelial nitric oxide synthase (eNOS) but decrease that of vascular cell adhesion molecule 1 (VCAM-1; Vazquez-Prieto et al., 2011). In rat isolated thoracic aorta, *A. cepa* (0.06–2.00 mg/ml) attenuated both phenylephrine- and KCl-induced contractions (Naseri et al., 2008). Removal of endothelium or inhibition of NO, cyclic guanosine monophosphate (cGMP), or prostaglandins did not affect the vasorelaxant action of onion (Naseri et al., 2008). These data suggest an endothelium-independent mechanism, possibly through the regulation of extracellular Ca^2+^ levels (Naseri et al., 2008). Indeed, the investigators implied that antioxidants and the polyphenol quercetin may play a role in relaxing the rat aorta (Naseri et al., 2008; Table 2). In contrast to the main conduit vessel, small resistance-size arteries of the rat mesentery, when exposed to a high cholesterol diet enriched with 10% onion powder, demonstrated an improved endothelium-dependent relaxation (comparable to the control group) than the impaired arterial relaxation observed with the group on high cholesterol diet alone (González-Peña et al., 2014). This improvement appears to be due to suppression of nicotinamide adenine dinucleotide phosphate (NADPH)-oxidase activity along with a concomitant increase in antioxidant kinetics of superoxide dismutase (SOD) and glutathione peroxidase (GPX) enzymes (González-Peña et al., 2014).

### *Alpinia zerumbet* (shel ginger)

*Alpinia zerumbet*, a west Asian plant, has been reported for its modest hypotensive effects (Lahlou et al., 2003; de Moura et al., 2005; Shen et al., 2012; da Cunha et al., 2013; Table 2). There is a growing consensus on its vasorelaxant responses, either through effects on endothelial cells (de Moura et al., 2005; Shen et al., 2012; da Cunha et al., 2013) or VSMCs (Lahlou et al., 2003). Specifically, the methanolic extract of its leaves (100 and 300 μg/ml) induced vasodilation by increasing NO or cGMP production in deoxycorticosterone acetate (DOCA)-salt-treated rats (de Moura et al., 2005). Interestingly, components of its essential oils (1–20 mg/kg) inhibit Ca^2+^ channels of rat thoracic aortic rings (da Cunha et al., 2013). In another study, 0.1 mg/L of *A. zerumbet's* essential oils were reported to reduce levels of oxidized low-density lipoprotein (LDL) in plasma, thereby highlighting their potential in preventing endothelial damage (Shen et al., 2012; Table 1).

### *Apocynum venetum* (dogbane or indian hemp)

Extracts of dogbane or Indian hemp's (common names) leaves have been reported to lower BP in different animal models (Ma and Chen, 1989; Kim et al., 2000; Lau et al., 2012; Xie et al., 2012; Table 2). Moreover, these extracts (10 μg/ml) induce vasorelaxation in rat aortic rings by increasing NO production and scavenging reactive oxygen species (ROS) (Lau et al., 2012; Table 1). Apparently, improvements in renal function are integral to the antihypertensive effect of this plant's extracts (Kim et al., 2000).

### *Arctium lappa* (burdock)

*Arctium lappa*, commonly known as burdock, is used for treatment of many ailments including hypertension (Lee Y. J. et al., 2012; Table 2). Evidence shows that this plant possesses ROS scavenging activity (Predes et al., 2011; Table 1), prevents vascular inflammation (Lee Y. J. et al., 2012; Wang et al., 2016; Table 3), and promotes vasorelaxation (Lee Y. J. et al., 2012; Table 2). Very recently, root extracts of this plant were shown to alleviate high fat diet-induced atherosclerotic lesions in quail (Wang et al., 2016). The underlying mechanisms for this protective effect appear to be due to the extracts' hypolipidemic and antioxidant capacities (Wang et al., 2016). Taken together, these activities could offer an explanation for the use of *A. lappa* as an antihypertensive agent.

One of the bioactive constituents found in dry seeds of burdock is arctigenin. It was recently found that arctigenin ameliorates endothelial dysfunction and reduces SBP in spontaneously hypertensive rats (SHRs; Liu Y. et al., 2015). The mechanism by which arctigenin seems to elicit its effects involves increased NO production as well as reduced levels of superoxide anion in thoracic aorta (Liu Y. et al., 2015). Interestingly, this bioactive ingredient was recently suggested to be a potential antihypertensive drug lead compound, owing to its newly discovered capacity as an antagonist of Mineralocorticoid receptor (Cheng et al., 2016). It is important to note here that although some report that this plant is popularly used for treatment of hypertension (Lee Y. J. et al., 2012), little evidence supports this notion; hence, we opted for including this plant among the herbs/plants that are less commonly used.

### *Avena sativa* (common oat)

The lowering of blood pressure (BP) by the common oat is still debatable (Houston, 2005; Kochar et al., 2012). While some studies report significant falls in BP (Houston, 2005; Flint et al., 2009; Kochar et al., 2012), others have failed to detect any change (Davy et al., 2002; Houston, 2005; Kochar et al., 2012). In a clinical study, an intake of low-calorie diet with oats [45 g dry weight/(4.2 MJ dietary energy. d)] for a 6-week period had a greater effect on reducing BP than one without oats in healthy men and women (Saltzman et al., 2001). Indeed, another clinical study reported that oat-containing diets decrease the necessity for hypotensive therapy (Houston, 2005).

The hypotensive effect of oats is attributable to the presence of its soluble fibers (Flint et al., 2009) and avenanthramides (biphenolic molecules; Andersson and Hellstrand, 2012). Mechanisms by which this effect is enacted suggests that it is due to several parameters, namely prevention of endothelial dysfunction (Guo et al., 2008; Flint et al., 2009; Kochar et al., 2012), decrease in TNF-α levels (Kochar et al., 2012), improved insulin sensitivity (Houston, 2005; Kochar et al., 2012), increased weight loss (Flint et al., 2009), inhibition of VSMC proliferation (Nie et al., 2006a,b), and increased NO production (Nie et al., 2006b). Following ingestion of oats, the soluble fiber β-glucan (linear, unbranched polysaccharide), is anaerobically broken-down by commensal microbiota into short-chain fatty acids such as butyrate, propionate and acetate (Andersson and Hellstrand, 2012). All of these compounds elicit arterial relaxation (Aaronson et al., 1996), which would cause a fall in BP, and their synergistic effects may prove to be even more significant.

### *Carthamus tinctorius* L. (safflower)

*Carthamus tinctorius* L. is used in traditional Chinese medicine for the treatment of cerebrovascular and cardiovascular diseases (Wang et al., 2014). The safflower extract is commonly referred to as safflower yellow (SY). SY is a mixture of water-soluble chalcone compounds extracted from the plant flowers (Zhou et al., 2013). It has been shown that SY lowers BP as well as reduces renin activity and angiotensin II levels in SHRs (Zhou et al., 2013). Hydroxysafflor yellow A (HSYA; 0.1–3 mg/kg), the main bioactive component of SY, has been shown to reduce BP and heart rate of both normotensive rats and SHRs (Nie et al., 2012; Table 2). Its mode of action appears to be elicited by opening K_ATP_ channels (Nie et al., 2012). Another report indicates that HSYA relaxes rat pulmonary artery by activating K_v_ channels (Bai et al., 2012). In addition to lowering BP in healthy humans, safflower seed extract (2.1 g daily) also decreased both VCAM-1 and LDL levels as well as reduced arterial stiffness (Koyama et al., 2009; Table 3). Additional evidence supports the regulatory effect of safflower on vascular tone in hypertensive rabbits (Di and Chang, 2007). Furthermore, two major indolic polyphenols in safflower seeds, namely N-(p-coumaroyl)serotonin (CS) and N-feruloylserotonin (FS), were shown to relax rat femoral arteries in an endothelium-independent manner (Takimoto et al., 2011). They also inhibited PDGF-induced proliferation of VSMCs (Takimoto et al., 2011). These data suggest a hypotensive effect of *C. tinctorius L*. that warrants further investigation. However, in a double-blind, placebo-controlled clinical trial, ingestion of ethanolic safflower extract (70 mg/day; for 12 weeks) caused a marginal yet not significant decrease in SBP or DBP (Suzuki et al., 2010). The study reported, however, that arterial stiffness and vascular aging were ameliorated (Suzuki et al., 2010; Table 6).

### *Cassia occidentalis* (reclassified as *senna occidentalis*) (coffee weed)

Coffee weed (common name) has been found to lower BP level (Ajagbonna et al., 2001), possibly due to inhibition of external Ca^2+^ influx via voltage-dependent channels (Ajagbonna et al., 2001; Table 2). In addition, aqueous extracts of the plant leaves (3.07 and 3.63 mg/ml) control vascular tone in rat aortic rings by mechanisms that were not affected by endothelium-denudation, indomethacin, or methylene blue (Ajagbonna et al., 2001). The aforementioned anti-hypertensive effects may be facilitated by other synergistic actions of *C. occidentalis*. For instance, coffee weed leaves possess anti-inflammatory and anti-oxidant properties, evident by their ability to reduce lipid peroxide content and suppress activity of phospholipase A_2_ (Yadav et al., 2010). Recently, it was also shown that the aqueous extract of this plant has a diuretic effect and improves kidney function indices in rats (Ntchapda et al., 2015).

### *Cinnamomum zeylanicum* (cinnamon)

Cinnamon is a known functional food. It has been reported to produce a blood lowering effect in several rat models (Nyadjeu et al., 2011, 2013; Table 2) as well as in type 2 diabetic and pre-diabetic humans (Akilen et al., 2013). The aqueous extract of its stem bark has been shown to reduce sucrose-induced elevation in SBP of SHRs (Preuss et al., 2006) as well as inhibit contractions induced by KCl in rat isolated aortic rings (Nyadjeu et al., 2011). This latter effect was dependent on the endothelium, NO, and K_ATP_ channels (Nyadjeu et al., 2011). Interestingly, the methanolic extract of the bark (300 mg/kg/day for 4 weeks) has also been shown to increase levels of NO in L-NAME-induced hypertension in rats (Nyadjeu et al., 2013). These data suggest a NO-dependent mechanism for the antihypertensive effects of *C. zeylanicum*. Importantly, during the revision of this manuscript, Azimi et al. published the results of a parallel randomized placebo-controlled clinical trial showing that consumption of cinnamon does not affect BP in type II diabetic patients (Azimi et al., 2016). However, this study suffered from many limitations including the short duration and small amount of cinnamon consumed, in addition to the fact that the sample participants were relatively old.

### *Cirsium japonicum* (japanese thistle)

*Cirsium japonicum* is a perennial herb that is typically found in China, Korea and Japan, and it belongs to the family Compositae. This Japanese thistle (common name) was suggested to be have an antihypertensive value (Kim et al., 2013). Its aqueous extract (0.1–1.0 mg/ml) has been reported to induce vasorelaxation of noradrenaline (0.3 μM) pre-constricted rat isolated thoracic aortic rings through activation of histamine H1 receptors (Kim et al., 2008). The underlying mechanism involves elevated levels of NO and cGMP (Kim et al., 2008; Table 2).

Thistles have also been used for the treatment of inflamed venular system, indicative of anti-inflammatory properties. Indeed, silibinin (0.05–0.4 mg/ml), a polyphenolic constituent of *C. japonicum*, inhibited the expression and release of inflammatory cytokines (TNF-α, IL-6, and IL-8), and decreased the NF-κB transcriptional capacity via diminishing the phosphorylation of IκBα (Kim et al., 2013; Table 3). More recently, silibinin was found to be an antagonist for the human angiotensin AT_1_ receptor (Bahem et al., 2015). This may provide a potential explanation for the observation that silibinin reduced SBP in L-NAME-treated rats (Souza et al., 2012).

### *Coleus forskohlii* (makandi)

Also known as Makandi, the *Coleus forskohlii* plant is central to Ayurvedic herbal remedies. This plant is famous for its isolate, forskolin, or coleonol (labdane diterpenoid), which is a potent adenylyl cyclase activator. Forskolin increases intracellular levels of cAMP leading to activation of protein kinase A (PKA, or cAMP-dependent protein kinase), which in turn induces relaxation of vascular smooth muscle cells (Lindner et al., 1978; Tirapelli et al., 2010; Table 2). Therefore, it is not surprising that forskolin has been reported for its BP lowering activity in cats (Dubey et al., 1981), rats (Dubey et al., 1981) including SHRs (Dubey et al., 1981), and humans (Dubey et al., 1981; Tirapelli et al., 2010; Table 2).

### *Cynanchum wilfordii* (dog-strangling vine)

*C. wilfordii* ethanolic extracts (100 and 200 mg/kg/day) are known to reduce BP in high fat/cholesterol-fed rats (Choi et al., 2012a; Table 2). This extract acts by activating Akt, leading to an enhanced eNOS activity, and release of increased levels of NO (Choi et al., 2012a). In ApoE-deficient mice on a high fat/cholesterol diet, *C. wilfordii* mitigated aortic endothelial dysfunction (Choi et al., 2012b). This was dependent on augmented NO and cGMP production, as well as decreased the expression of VCAM-1 and ET-1 (Choi et al., 2012b; Tables 2, 3). Indeed, it was previously shown that *C. wilfordii* induces endothelium-dependent and cGMP-mediated relaxation of rat aorta (Choi et al., 2010). These results suggest a robust hypotensive response of *C. willfordii* in experimental rodent models.

### *Echinodorus grandiflorus* (burhead)

*Echinodorus grandiflorus* (Cham. & Schltdl.) Micheli is a semi-aquatic plant that is native to Brazil. This plant belongs to the Alismataceae family. Evidence suggests that the aqueous extracts of this plant *have* the ability to reduce MAP as well as cardiac output and vascular resistance in SHRs (Lessa et al., 2008; Table 2). The crude extract has also been reported to induce relaxation of endothelium-intact rabbit isolated aortic rings (Tibiriçá et al., 2007); these effects were reversed when NO production was inhibited (Tibiriçá et al., 2007; Lessa et al., 2008). This implies that *E. grandiflorus*'s cardioprotective, vasorelaxant and hypotensive effects are modulated by an NO-dependent mechanism. While this manuscript was being reviewed, Prando et al. reported that the ethanolic extract of *E. grandiflorus* causes a hypotensive as well as antihypertensive effect in a renovascular rat model of hypertension, two-kidney-one-clip (2K-1C; Prando et al., 2015). These two effects appear to be modulated by bradykinin B_2_ and muscarinic receptors as well as NO and cyclooxygenases (Prando et al., 2015).

### *Elettaria cardamomum* (cardamom)

*Elettaria cardamomum* is commonly known as cardamom. In powder form (3 g), it decreases mean arterial blood pressure (MAP), as well as SBP and DBP in pre-hypertensive (Stage 1) subjects (Verma et al., 2009; Table 6). SBP and DBP were significantly decreased by 19 and 12 mmHg, respectively (Verma et al., 2009). The mechanism for this hypotensive action appears to be due to cardamom's ability to increase the total antioxidant status (Verma et al., 2009; Table 1). In anesthetized rats, 3–100 mg/kg of *E. cardamomum* crude extract was also able to reduce BP (Gilani et al., 2008). In the same model, 1–10 mg/kg of the crude extract exhibited diuretic effects (Gilani et al., 2008; Table 5). It also relaxed pre-constricted rat aortic rings with a concentration of 2.94 mg/ml, possibly by inhibiting Ca^2+^ movement through transmembrane calcium channels (Gilani et al., 2008; Table 2). Interestingly, during the revision of this manuscript, Azimi et al. reported that they failed to find any appreciable effect of cardamom or BP in type II diabetic patients (Azimi et al., 2016).

### Embelia ribes

*Embelia ribes* has been reported to produce different hypotensive effects. In isoproterenol-treated rats, the aqueous extract of *E. ribes* (100 mg/kg) was able to decrease both the SBP and heart rate, as well as increase endogenous antioxidants such as SOD, CAT, and GSH (Bhandari et al., 2008a,b) (Table 1). Similar results were produced after treatment of high fat-fed rats with 50 mg/kg of embelin, a bioactive component of the herb (Chaudhari et al., 2012; Table 1).

### *Gastrodia elata blume* (tianma)

*Gastrodia* rhizome has been reported to possess antihypertensive effects (Lee O. H. et al., 2012). Treatment of high fat-fed SHR with acidic polysaccharides isolated from the rhizome (6 mg/kg of body weight/day for 5 weeks) caused a significant reduction in BP level (Lee O. H. et al., 2012). In the same study, a decrease in the concentration of total cholesterol, LDL-cholesterol, and triglycerides in serum were also noted (Lee O. H. et al., 2012; Table 1). In addition, the methanolic extracts (0.02 ml/g) of *Gastrodia* rhizome showed anti-inflammatory effects by reducing iNOS expression, and thereby decreasing the level of NO (An et al., 2007; Table 3). There are other reports that show the beneficial cardiovascular effects of gastrodin, a major bioactive component of *Gastordia elata* Bl. For example, injection of gastrodin into elderly patients with refractory hypertension caused a decrease in systolic and pulse pressures (Zhang et al., 2008). Furthermore, this study showed that gastrodin increase NO levels while it simultaneously reduced endothelin levels (Zhang et al., 2008). Recently, the underlying mechanism for gastrodin's effects was elucidated. Liu et al. showed that gastrodin lower SBP by interfering with the Renin-Angiotensin-Aldosterone system (RAAS) (for further explanation on the role of RAAS, please refer to Part I of this review Al Disi et al., 2015). Indeed, gastrodin decreased serum levels of Ang II as well as expression of both of ACE and AT_1_R (Liu W. et al., 2015).

### Gentiana floribunda

Recently, *Gentiana floribunda's* BP lowering effect was reported (Khan et al., 2012; Table 2). It relaxed the aorta of rats in a dose-dependent manner (0.3–1 mg/ml), possibly by blocking Ca^2+^ channels (Khan et al., 2012). L-NAME did not affect *G. floribunda's* activity, indicative of a NO-independent mechanism (Khan et al., 2012).

### *Gossypium barbadense* (pima cotton)

Commonly known as pima cotton, *G. barbadense* induces relaxation of guinea pig aorta, which may offer an explanation for its observed lowering effect on BP (Hasrat et al., 2004; Tabassum and Ahmad, 2011).

### *Gynura procumbens* (pointed phoenix tail)

The aqueous extract of *Gynura procumbens* has been reported to produce blood-pressure-lowering effect in SHRs (Kim et al., 2006; Hoe et al., 2007) and in WKY (Hoe et al., 2007; Table 2). It can also inhibit Ang I- and Ang II-induced contractions in rat aortic rings via a NO-dependent mechanism (Poh et al., 2013). This result is in agreement with other reports showing the ability of 500 mg/kg of *G. procumbens* extract to increase levels of NO in SHRs (Kim et al., 2006). The aqueous extract has also been reported to inhibit ACE activity (Poh et al., 2013). Furthermore, a partially purified fraction of *G. procumbens* inhibited Ang I-induced elevation in MAP, apparently via inhibiting ACE activity (Hoe et al., 2007).

The crude extract (0.003 and 0.009 g/ml) also exhibited vasodilatory effect, as it inhibited both KCl- and phenylephrine-induced contractions in rat isolated thoracic aortic rings (Ng et al., 2013). This vasorelaxant function is related to the opening of potassium channels, inhibition of calcium channels and release of prostacyclin (Ng et al., 2013). All these findings allude to the beneficial role of *G. procumbens* as a hypotensive agent.

### *Kaempferia parviflora* (thai or black ginseng)

Not only can *Kaempferia parviflora*, or black ginseng, inhibit phenylephrine-induced contraction of rat isolated aortic rings (Wattanapitayakul et al., 2008), but it can also induce their relaxation (Tep-Areenan et al., 2010; Table 2). This relaxation is due to *K. parviflora's* bioactive component, 5,7- dimethoxyflavone (1–100 μM), which augmented NO and cGMP levels (Tep-Areenan et al., 2010). The same study reported that 10 and 100 μM of 5,7-dimethoxyflavone exerts vasorelaxant effects by reducing extracellular Ca^2+^ influx (Tep-Areenan et al., 2010). The same vasorelaxant mechanisms were also reported for this herb in rat hearts and ventricular myocytes (Weerateerangkul et al., 2012; Table 2).

It is important to note that other mechanisms may also contribute to black ginseng's antihypertensive potential. For example, this plant was shown to potently reduce oxidative stress in aortae of diabetic rats (Malakul et al., 2011). This is in line with a more recent report showing that extracts of *K. parviflora* ameliorate endothelial dysfunction by reducing oxidative stress and increasing eNOS in human endothelial cells (Wattanapitayakul et al., 2007; Horigome et al., 2015). These extracts also inhibited adhesion of monocytes to endothelial cells as well as reduced levels of pro-inflammatory cytokines (Horigome et al., 2015).

### *Lavandula stoechas* (lavender)

French lavender extracts cause decreases in heart rate and BP in rats (Tabassum and Ahmad, 2011). They were also reported to relax rabbit intestines (0.1–1 mg/ml), possibly by Ca^2+^ channel inhibition (Gilani et al., 2000; Table 2). This relaxant activity on intestines' smooth muscles could indicate hypotensive effect if confirmed on VSMCs or endothelial cells. However, it remains to be determined. Constituents of the Lavendula genus are known to reduce inflammatory stress through different mechanisms such as scavenging free radical, promoting antioxidants levels, and reducing the concentration of arachidonic acid metabolites (Sosa et al., 2005).

### Lepidium

A hypotensive effect of *Lepidium sativum* (garden cress; 20 mg/kg) has been reported in SHR but not WKY rats (Maghrani et al., 2005). This effect appears to be related to the increased urinary elimination of sodium, potassium, and chlorides (Maghrani et al., 2005; Table 5). Recently, two new glycosides isolated from seeds of *L. sativum* were shown to have an anti-inflammatory potential (Fan et al., 2014). Another member of the Lepidium genus is *Lepidium latifolium*, commonly known as broadleaved pepper weed. This plant has been shown to induce diuresis (Navarro et al., 1994), an effect that has been observed in both rats and man (Navarro et al., 1994; Wright et al., 2007; Table 5). Moreover, *Lepidium latifolium* displays a potent antioxidant capacity, which may contribute to its antihypertensive effects (Kaur et al., 2013; Table 1).

### *Marrubium vulgare* (white horehound)

White horehound is reported to significantly decrease SBP in SHRs (El Bardai et al., 2001, 2004; Table 2). This hypotensive effect is probably due to its vasorelaxant and anti-hypertrophic activities. White horehound was found to relax rat aorta (El Bardai et al., 2001), potentiate acetylcholine (ACh)-induced relaxation of mesenteric artery (El Bardai et al., 2004; Table 2), and elicit an antihypertrophic effect in aorta (El Bardai et al., 2004). Importantly, this plant can also ameliorate the impaired endothelial function in SHRs (El Bardai et al., 2004). The mechanism of action is not yet completely understood, but it appears to be NO-independent as L-NAME did not affect horehound's vasodilatory action (El Bardai et al., 2001).

Different molecules isolated from white horehound seem to contribute to these vasculoprotective effects. For example, the diterpene marrubenol can potently block L-type calcium channels and consequently inhibit contraction of VSMCs (El-Bardai et al., 2003). Both marrubenol and marrubiin, another diterpene from *Marrubium vulgare* inhibit KCl-induced contractions of rat aorta (El Bardai et al., 2003). Furthermore, phenylpropanoids isolated from this plant can also inhibit lipoprotein-induced secretion of the potent vasoconstrictor endothelin-1 (Martin-Nizard et al., 2004).

### *Melothria maderaspatana* (melon-gubat)

*M. maderaspatana* has been reported to diminish BP in DOCA-salt hypertensive rats (Veeramani et al., 2011, 2012; Table 2) as well as in hypertensive humans (Raja et al., 2007). Indeed, individuals with mild hypertension that consumed melon-gubat tea (4% w/v leaf powder) for 45 days showed a significant decrease in both SBP and DBP (23.8 and 15.5 mm Hg, respectively) (Raja et al., 2007; Table 6). It has also shown a positive effect on eNOS (Veeramani et al., 2012) and cellular antioxidants (Veeramani et al., 2011) levels in DOCA-salt induced hypertension (Table 1). Importantly, *M. maderaspatana* seems to decrease levels of ET-1, epinephrine and norepinephrine (Veeramani et al., 2012).

### *Ocimum basilicum* (sweet basil)

Sweet basil's crude extract dose-dependently (100–400 mg/kg) decreased BP level in rats (Umar et al., 2010), as well as inhibited renovascular hypertension-induced hypertrophy of heart and increase in ET-1 and Ang II levels (Umar et al., 2010; Table 2). It was also reported to cause a vasorelaxant effect in rat aortic rings, though the mechanism for this relaxation was not determined (Amrani et al., 2009; Table 2). Perhaps one potential mechanism could be due to the sweet basil's potent ROS scavenging ability (Kaurinovic et al., 2011; Table 1).

### *Peganum harmala* (esfand)

*Peganum harmala* is commonly known as Esfand and is used in some cultures for the treatment of hypertension (Tahraoui et al., 2007). Not only does *P. harmala* inhibit the contraction of rat isolated aorta (Astulla et al., 2008), but it also induces relaxation via both endothelial cells and VSMCs (Shi et al., 2001; Astulla et al., 2008; Table 2). Interestingly, Berrougui et al. report that the vasorelaxant effect of *P. harmala* seeds' methanolic extract is endothelium-independent (Berrougui et al., 2002). Esfand's active components—harmine, harmaline, and harmalol (3–30 μM)—exert a vasodilatory effect that is thought to be a result of its ability to increase NO production (Shi et al., 2001; Berrougui et al., 2006; Table 2). The Quinazoline alkaloid, vasicinone, isolated from seeds of Esfand is also reported to induce vasorelaxation in phenylephrine-contracted rat aorta (Astulla et al., 2008).

### *Phyllanthus amarus* (stonebreaker; seed-under-leaf)

The stonebreaker is reported to decrease BP in rabbits (Amaechina and Omogbai, 2007) and humans (Srividya and Periwal, 1995). This antihypertensive effect is likely related to its diuretic, antioxidant, and anti-inflammatory activities (Kassuya et al., 2005; Maity et al., 2013; Tables 1, 3, 5). In a clinical trial, *P. amarus* increased urinary output as well as levels of Na^+^ in urine (Srividya and Periwal, 1995). These actions were without any noticeable harmful side effects in the mild hypertensive subjects of the study (Srividya and Periwal, 1995). In addition, the aqueous extract of *P. amarus* (200 mg/kg) was shown to increase plasma antioxidants (GSH, GPx, SOD, and CAT) in rats (Karuna et al., 2009; Table 1). Furthermore, different solvent extracts of *P. amarus* have been reported to inhibit of NF-κB, TNF-α, and COX-2 in LPS-treated RAW 264.7 macrophages (Kiemer et al., 2003; Table 3).

### *Pueraria lobata* (kudzu)

*Pueraria lobata's*, or gegen's, isoflavones are reported to decrease BP in dogs and in hypertensive patients (Tabassum and Ahmad, 2011). This is possibly due to gegen's vasodilatory effect, which could be a result of its ability to activate calcium-activated potassium channels in rat thoracic aorta (Sun et al., 2007; Ng et al., 2011; Table 2). However, when combined with *Salvia miltiorrhiza, P. lobata* offered cardiovascular protective effects in high-risk hypertensive patients (Woo et al., 2013). This adjunctive treatment was able to improve endothelium-dependent vasodilation as well as decrease carotid intima-to-media thickness (Woo et al., 2013). *P. lobata*'s anti-inflammatory and anti-oxidant activities may partly explain the anti-hypertensive effects of this plant (Jin et al., 2012; Tables 1, 3).

Puerarin [7-hydroxy-3-(4-hydroxyphenyl)-1-benzopyran-4-one 8-(β-D-glucopyranoside)] is the major bioactive compound isolated from kudzu. It is reported that puerarin possesses antihypertensive and other cardioprotective effects (Song et al., 1988; Li et al., 2008). Moreover, puerarin could be beneficial in the management of hypoxia-induced pulmonary hypertension (Chen et al., 2011, 2012; Zhang et al., 2011).

### *Raphanus sativus* (radish)

Extracts of seeds or leaves of radish have been reported to induce BP-lowering effect in rats. In SHRs, leave extracts (30 and 90 mg/kg) reduced SBP (Chung et al., 2012), while the seeds (0.1–3 mg/kg) lowered BP as well as the heart rate (Ghayur and Gilani, 2006; Table 2). In SHRs, radish extracts were also reported to enhance NO production and increase antioxidant levels (Chung et al., 2012; Table 1). The seed extract (0.3–3 mg/ml) exhibited vasorelaxant effects, and was able to inhibit contraction of rat aorta as well as atria of guinea pigs (Ghayur and Gilani, 2006). These vasorelaxant effects of radish are thought to be mediated by NO production (Ghayur and Gilani, 2006). In addition, both methanolic and acetone extracts of radish were able to scavenge ROS in an enzymatic assay (Beevi et al., 2010). The anti-proliferative and anti-inflammatory capacities of radish may also be partial contributors to its overall antihypertensive effects. Indeed, radishes can inhibit proliferation of VSMCs and arrest them in G1 phase of the cell cycle (Suh et al., 2006; Table 4). Radish can also inhibit LPS-induced pro-inflammatory molecules such as NO, IL-1β, TNF-α, and IFN-γ (Kook et al., 2014; Table 3). Recently, it was also shown that phenylpropanoid sucrosides isolated from seeds of radish can also inhibit LPS-induced inflammation in murine BV-2 microglial cells (Kim et al., 2015). For more details on role of inflammation or VSMC proliferation in hypertension, part I of this review could be referred to (Al Disi et al., 2015).

### *Rhazya stricta* (harmal)

*Rhazya stricta's* leaves have been documented to lower BP in rats (Tanira et al., 2000; Table 2). In addition, Tanira et al. reported that *R. stricta* induced relaxation in guinea pig and rabbit small intestines (Tanira et al., 1996).

### *Rhus coriaria* (sumac)

*Rhus coriaria*, commonly known as sumac, is known for its antioxidant activity (Candan and Sokmen, 2004; Kosar et al., 2007; Pourahmad et al., 2010; Table 1). Extracts of this plant's leaves have been shown to cause relaxation of isolated rabbit aorta rings pre-contracted with norepinephrine (Beretta et al., 2009; Table 2). This vasorelaxant effect appears to be predominantly endothelium-dependent and NO-mediated (Beretta et al., 2009). Moreover, inhibition of cyclooxygenase (COX) by indomethacin significantly diminished sumac's vasodilatory effect, clearly suggestive of a role for COX enzymes in the observed vasorelaxation (Beretta et al., 2009). Importantly, this extract also possesses a potent anti-inflammatory capacity, evident by its ability to reduce ischemia-induced TNF-α (Beretta et al., 2009).

### *Sesamum indicum* (sesame)

Sesame is known to decrease BP as well as the heart rate in rats (Tabassum and Ahmad, 2011). Petroleum ether soluble fraction of root extract of sesame plant induced vasorelaxation, dose-dependently up to a 180 mg/ml, in rat isolated aorta (Suresh Kumar et al., 2008). Introducing an eNOS inhibitor, L-NAME, or removal of the endothelium decreased the extract's vasodilatory capacity (Suresh Kumar et al., 2008), indicative of a mechanism that relies on both the endothelium and NO generation (Table 2). An ethanolic seed extract of *S. indicum* also exhibits antioxidant activity, as it scavenges ROS, inhibits LDL peroxidation (Visavadiya et al., 2009) and enhances the kinetic properties of antioxidant enzymes (Tabassum and Ahmad, 2011; Table 1). Furthermore, in a randomized clinical trial among type 2 diabetics, patients consuming a paste of ground unhulled sesame seeds (28 g/day) exhibited a favorable modulation of several CVD risk factors, particularly a reduction in serum levels of triglycerides, (Mirmiran et al., 2013).

### *Solanum* (tomato)

*Solanum sisymbriifolium* (wild tomato) and *Solanum paludosum Moric* (SpM) exhibit hypotensive effects. Root extract of wild tomatoes is reported to decrease BP level in normotensive and hypertensive rats (Ibarrola et al., 1996, 2000, 2011). *SpM* (75 μg/ml) exhibits vasodilatory activities in rat aortic rings by a NO-dependent pathway (Monteiro et al., 2012; Table 2). In a double-blind, placebo-controlled pilot study, an antioxidant-rich tomato extract was shown to reduce both systolic and diastolic BP in grade-1 hypertension subjects (Engelhard et al., 2006). Importantly, tomato extract significantly lowered BP in patients with hypertension controlled with low doses of calcium-channel blockers or ACE inhibitors (Paran et al., 2009). In a randomized control trial, tomato extract was not found to have a BP-lowering effect in a pre-hypertensive population (Ried et al., 2009).

### *Stephania tetrandra* (stephania root; fang ji, etc.)

*Stephania tetrandra* was able to normalize elevated BP in rats (Wong et al., 2000; Yu et al., 2004), as well as decrease heart rate (Wong et al., 2000) and inhibit hypertrophy (Yu et al., 2004). It can also relax rabbit penis (corpus cavernosum), possibly by reducing Ca^2+^-induced muscle contraction (Qian et al., 1983; Chen et al., 2009; Table 2). The main bioactive component of this plant is tetrandrine. Tetrandrine is reported to have anti-inflammatory (Ferrante et al., 1990; Chen et al., 1997; Xie et al., 2002; Wu and Ng, 2007; Dang et al., 2014; Table 3) and anti-oxidant (Shi et al., 1995; Table 1) properties, both of which likely contribute to the antihypertensive effects of this plant.

### *Tribulus terrestris* (bindii, cat's head, devil's eyelashes, etc.)

*Tribulus terrestris*, or Bindii, is reported to reduce BP in SHR (Phillips et al., 2006) and two-kidney one-clip (2K-1C) rats (Sharifi et al., 2003). In the 2K-1C rats, 10 mg/kg of Bindii also reduced ACE activity (Sharifi et al., 2003). The methanolic and aqueous extracts (0.3–15 mg/ml) exhibited vasodilatory effects in SHRs via a mechanism that appears to involve both NO release and membrane hyperpolarization (Phillips et al., 2006; Table 2). This plant is also reported to be used for its diuretic value in Indian folk medicine (Kumar et al., 2015). Moreover, total saponins of this plant inhibit Ang II-induced production of H_2_O_2_ (Table 1) as well as proliferation of VSMCs induced by Ang II (Li et al., 2013; Table 4).

### *Tropaeolum majus L*. (garden nasturtium, monks cress, etc.)

Hydroethanolic extracts of *Tropaeloum majus* have shown a concentration-dependent reduction in MAP of SHR and normotensive rats (Gasparotto Junior et al., 2011) (Table 2). This hypotensive effect could be a result of different mechanisms. For instance, *T. majus'* ethanolic extract (300 mg/kg), purified fraction (100 mg/kg), or isoquercitrin (10 mg/kg), the plant's main component, are all known to exhibit diuretic activities in SHR (Gasparotto Junior et al., 2012; Table 5). This is thought to be a result of their ability to increase plasma aldosterone levels and downregulate sodium/potassium pump activities in the kidney (Gasparotto Junior et al., 2012). All the aforementioned components were able to reduce plasma ACE levels (Gasparotto Junior et al., 2011, 2012). Isoquercitrin, in particular, has also been reported to decrease ROS levels and increase NO production (Gasparotto Junior et al., 2012; Table 1).

### *Viola odorata* (sweet violet)

Sweet violet leaves extract (0.1, 0.3, and 1 mg/kg) is reported to lower MAP of rats (Siddiqi et al., 2012; Table 2). The extract induced relaxation and also caused a rightward shift of Ca^2+^ concentration-response in a concentration-dependent (0.3–3 mg/ml) fashion (Siddiqi et al., 2012). This vasodilatory effect is thought to be mediated through NO production and Ca^2+^ influx control (Siddiqi et al., 2012). Relevantly, the extract favorably ameliorated CVD risk factors by causing a significant decrease in total cholesterol, LDL-C, and atherogenic index (Siddiqi et al., 2012). It also increased HDL-C levels, and prevented a body weight increase in Sprague-Dawley rats (Siddiqi et al., 2012).

### Viscum articulatum burm

A traditional Chinese herb, *Viscum articulatum Brum*, has been used for treatment of hypertension. It has been reported to have diuretic activities, as its methanolic extract increased urine volume of normal (Jadhav et al., 2010) and L-NAME-treated rats (Bachhav et al., 2012; Table 5). In addition, oleanoic acid isolated from *V. articulatum Brum* (60 mg/kg/day) can increase plasma NO levels, while also decreasing both lipid peroxidation in the heart and glucocorticoid-induced SBP (Bachhav et al., 2011; Tables 1, 2). An antioxidant potential of this plant has also been reported (Kuo et al., 2010; Table 1).

## Other less-studied plants with hypotensive activities

There are a considerable number of other plants that have been reported to induce hypotensive responses in different animal models of hypertension. Here we briefly discuss the ones that are not used as frequently as the above-mentioned plants and are not that well-studied.

*Cnidium monnieri*'s osthol has been reported to decrease BP of both renovascular hypertensive rats (20 mg/kg; Zhou et al., 2012) and stroke-prone SHRs (0.05% by weight; Ogawa et al., 2007), possibly through increasing antioxidant levels (Zhou et al., 2012; Table 1). Administration of an aqueous extract of *Cuminum cyminum* seeds (200 mg/kg body weight for 9 weeks) to 2K-1C rats also exhibits a BP-lowering effect. This fall in SBP was reported to arise from improvement in endothelial function reflected by upregulation of eNOS and hence augmented NO production, increase in expression of antioxidant system (thioredoxin 1 and thioredoxin reductase 1), and downregulation of inflammatory markers such as TNF-α and IL-6 (Kalaivani et al., 2013; Tables 1, 3).

*Kalanchoe pinnata* aqueous extract (25–100 mg/kg/day) has been reported to also decrease BP and exhibit antioxidant activities in high salt-loaded rats (Bopda et al., 2014; Table 1). *Peristrophe roxburghiana* (Cheng et al., 2004) and *Ranunculus japoniucus* (Liu et al., 2012; Table 2) was able to lower BP of 2K-1C rats, possibly through regulating NO and Ang II levels, respectively. *Picrasma quassiodes* has shown a hypotensive effect on SBP of SHR, which could be possibly related to modulating NO and SOD production (Zhao et al., 2013; Table 1). *Scrophularia ningpoensis* has shown an ameliorative effect on BP and heart rate of hypertensive rats, an effect that is possibly due to its ability to reduce levels of SOD and ET-1 (Gu and Chen, 2008).

*Lithocarpus polystachyus Rehd* (Hou et al., 2012) and *Thymus serpyllum* (Mihailovic-Stanojevic et al., 2013) were shown to decrease BP in SHR, while *Thymelaea hirsuta* has decreased BP of diabetic-hypertensive rats (Bnouham et al., 2012). *Pueraria tuberosa* is reported to reduce BP of stage 1 hypertensive humans (Verma et al., 2012). A mechanism for the hypotensive effect of these four herbs has not yet been elucidated.

Other plants may have potential hypotensive effect due to their effects on VSMCs. A mixture of *Angelica sinensis* and *Ligusticum chuanxiong* extract (300 μg/ml) has been reported to inhibit VSMCs proliferation by inducing a G0/G1 arrest (Hou et al., 2005; Table 4). *Artemisia verlotorum* Lamotte has shown a vasodilatory effect on isolated rat aorta, probably mediated via muscarinic receptor agonism and transduced through the NO/cGMP route (Calderone et al., 1999; Table 2). *Bacopa monnieri* also showed an NO-mediated vasodilatory effect on several types of blood vessels isolated from rats as well as decreasing BP (Kamkaew et al., 2011; Table 2). Its vasodilatory effect could also be a result of regulating Ca^2+^ levels (Kamkaew et al., 2011). A similar mechanism was also suggested for the vasorelaxant effect of an extract of *Crotalaria sessiliflora* (0.5–5 mg/ml) on rat thoracic aortic rings, where removal of endothelium, extracellular Ca^2+^ or inhibition with L-NAME abolished the relaxation of this conduit artery (Koh et al., 2007; Table 2). *Euphorbia humifusa* also elicited a vasodilatory effect on phenylephrine-induced contractions in rat aortic rings by modulating NO and Ca^2+^ levels (Wang et al., 2013; Table 2). *Geum japonicum*'s extracts (acetone and butyl alcohol—30 μg/ml) have been reported to lower BP in normotensive and hypertensive rats, and relax phenylephrine-induced contractions of rat aortic rings, while not affecting baseline tonicity, suggesting a normalizing role (Xie et al., 2007; Table 2). The mechanism for this plant appears to be dependent on both NO and cGMP production (Xie et al., 2007). Extracts from the roots of *Microdesmis keayana* (0.5–50 mg/ml) have shown ability to vasorelax aortic strips from normotensive rats and guinea pigs possibly by increased expression of eNOS (Zamble ´ et al., 2006; Table 2). Diterpenes isolated from *Moldenhawera nutans* (1–10 mg/kg) have been able to reduce contractions induced by high potassium in isolated rat aorta, apparently through regulation of sympathetic tone (Lahlou et al., 2007; Table 2).

Several herbs have also been reported to inhibit contractions in blood vessels through an endothelium-dependent mechanism via enhancing NO production (Table 2). These include: *Nitraria sibirica Pall* (0.1–10 g/l, Senejoux et al., 2012); *Oenothera odorata* (5.16 μg/ml, Kim et al., 2011); *Prunella vulgaris* (100 and 200 mg/kg, Hwang et al., 2012)**;** *Pseuderanthemum palatiferum* (81 μg/ml, Khonsung et al., 2011)**;** *Peucedanum praeruptorum Dunn* (1–100 μM, Xu et al., 2010); *Piper truncatum* (10.7 μg/ml, Raimundo et al., 2009); *Uncariae ramulus et Uncus* (Goto et al., 1999); and *Zizyphi Spinosi* (Fu et al., 2011). Furthermore, *Piper truncatum* also seems to mediate its effect via histamine receptors (Raimundo et al., 2009). In addition to vasodilation, *Peucedanum praeruptorum Dunn* has also been reported to decrease smooth muscle size and collagen content (Xu et al., 2010). On the other hand, a couple of herbs' vasorelaxant effect is mediated by Ca^2+^ regulation. *Petasites formosanus* (10–100 μM, Wang et al., 2002); and *Piper nigrum* (1–30 μM, Taqvi et al., 2008); are reported to antagonize Ca^2+^ channels and produce vasodilation in blood vessels. Finally, *Tridax procumbens* (0.15–1.05 mg/ml) has been reported to relax norepinephrine, KCl and serotonin-induced contractions in rat isolated aortic rings (Salahdeen and Murtala, 2012; Table 2); its mechanism has not yet been deciphered. Further *in vivo* investigations should be conducted to study and confirm the effect of these herbs and spices on hypertension.

Many other herbs and spices are reported to exhibit antioxidant activities. *Radix Angelicae* has been reported to increase cellular antioxidants, decrease proxidants and Ang II levels in 2K-1C rats (Cao et al., 2013; Tables 1, 2). *Ferula gummosa* (90 mg/kg; Gholitabar and Roshan, 2013) and *Sambucus ebulus L*. (200 ml/day) (Ivanova et al., 2014) were able to increase antioxidant enzymes in SHRs and healthy humans, respectively (Table 1). On the other hand, *Desmodium gangeticum* (Sankar et al., 2013) has been shown to reduce ROS levels in cardiomyocytes (Table 1). *Cnidium officinale* (Jeong et al., 2009) and *Suaeda asparagoides* (Park et al., 2007) possess antioxidant properties as confirmed by the DPPH enzymatic assay (Table 1). These antioxidant activities indicate a potential role in hypertension treatment that should be explored further for vasorelaxant activity.

Finally, several herbs and spices have been reported to demonstrate hypotensive properties. For example, *Radix astragali* regulates expression of eNOS and ET-1, as well as inhibit vascular remodeling in pulmonary arteries (He et al., 1999). *Sophora flavescens Ait* (0.5–2 mg/ml) has been reported to reduce expression of IL-6, VCAM-1, and ET-1 in addition to inhibiting proliferation of VSMCs in monocrotaline-induced pulmonary hypertension in rats (Zhang et al., 2014; Tables 3, 4). *Sorbus cortex* (100 and 200 mg/kg) is reported to regulate NO and ET-1, as well as decrease inflammatory markers (NF-κB, VCAM-1, ICAM-1) and media/lumen ratio in rats fed an atherogenic diet (Sohn et al., 2005; Tables 2–4). *Rheum rhabarbarum*'s aqueous extracts decreased ET-1, VCAM-1, and LDL levels (Moon et al., 2008). *Scutellariae radix* increased NO levels (Chen et al., 2013; Table 3).

## Epigenetic alterations in hypertension, and correction by consumption of herbs and their constituents

Three decades have elapsed since an association between epigenetic modification (DNA methylation) and disease was first recognized (Feinberg and Vogelstein, 1983). Subsequently, this has been further expanded through research into chromatin and histone modifications, and RNA-driven mechanisms (micro RNAs). These molecular factors are involved in the modification of the genetic material, without any alteration in the nucleotide sequence. Interestingly, cardiovascular pathological processes such as oxidative stress, proliferation, and inflammation can be ameliorated by preventing hypermethylation and regulation of epigenetic balance between histone acetyl transferases (HAT) and histone deacetylases (HDAC)/lysine deacetylases (KDACs; Millis, 2011; Sunagawa et al., 2011; Baccarelli and Ghosh, 2012; Stefanska et al., 2012).

### Epigenetic alterations

Accumulating data provides clear evidence that epigenetic processes play a significant role in many CVDs, including atherosclerosis (Turunen et al., 2009) and hypertension (Millis, 2011; Cowley et al., 2012; Yang et al., 2015). One of the most common epigenetic modifications is DNA methylation. Importantly, this modification plays vital roles in the regulation of transcriptional machinery and is thus implicated in several diseases, including CVDs (Bjornsson et al., 2004; Feinberg, 2008). Unlike DNA base sequences, these epigenetic modifications are potentially reversible, making them attractive targets in modern or personalized medicine.

The degree of eNOS expression is a robust biomarker for vascular tone and BP regulation. One of the mechanisms by which eNOS expression is regulated takes place via epigenetic mechanisms. For instance, oxidized LDL increases Histone 3 Lysine 9 (H3K9) methylation of eNOS promoter in endothelial cells (Fang et al., 2011). It also concurrently decreases acetylation of H3 and H4 as well as methylation of Histone 3 Lysine 4 (H3K4) in the proximity of eNOS promoter (Fang et al., 2011). Together, these findings are indicative of down-regulation of eNOS expression and thus a decrease in the bioavailability of NO (Fang et al., 2011). Moreover, in H3K4/K9 demethylase (LSD1)-deficient mice, eNOS expression is diminished, further implicating the role of histone methylation in vascular tone (Fish et al., 2010).

As is well known, the kidney plays an integral role in regulating systemic blood pressure. Notably, kidney functions appear to be significantly affected by the individual's global methylation profile (Beckerman et al., 2014; Wing et al., 2014).

The role of histone modifications in vascular tone is also evident in the regulation of endothelin (Yu et al., 2015), the most potent natural vasoconstrictor. There is increasing evidence to support the notion that endothelin promoter transactivation may be regulated by epigenetic mechanisms. Indeed, an increase in the histone acetyltransferase p300 has been shown to be accompanied with increased release of endothelin (Chiu et al., 2009). Another relevant observation is that treatment of endothelial cells with pro-inflammatory stimuli leads to increased H4 acetylation in the endothelin promoter (Wort et al., 2009). Moreover, epigenetic modulation appears to be an important contributor to endothelin-induced expression of pro-inflammatory genes in VSMCs (Yang et al., 2014). Interestingly, epigenetic regulation of endothelin can also be seen in other CVDs like cardiac hypertrophy (Czubryt, 2015).

### Nutrients deficient in methyl donors

Diets deficient in methyl donors, like betaine, choline, folate, and methionine are a pointer to dysregulation of metabolic and cardiovascular function (Stefanska et al., 2012). This is mainly caused by disturbances in DNA hypomethylation, plasma homocysteinemia, increases in S-adenosylhomocysteine (SAH), reduction in S-adenosylmethionine (methyl donor), and an unfavorable plasma lipid composition (Stefanska et al., 2012). This milieu can be considered as a contributory risk factor for the pathogenesis of cardiovascular diseases (Zawada et al., 2014). Notably, ingestion of phytochemicals could be very beneficial in adjusting the level of hypomethylation (Stefanska et al., 2012). Folate acts as a methyl donor and is important for synthesis and regulation of DNA. Indeed, a diet containing low folate content prior to pregnancy has a bearing on the future offspring and its health into adult-life, causing an obese phenotype and increasing the risk of hypertension (Sinclair et al., 2007). In contrast, a methyl-rich diet during pregnancy protects the offspring from developing the obese phenotype (Dolinoy et al., 2006).

### Involvement of herb- and plant-derivatives in the modulation of epigenetic machinery

**Figure 1:**
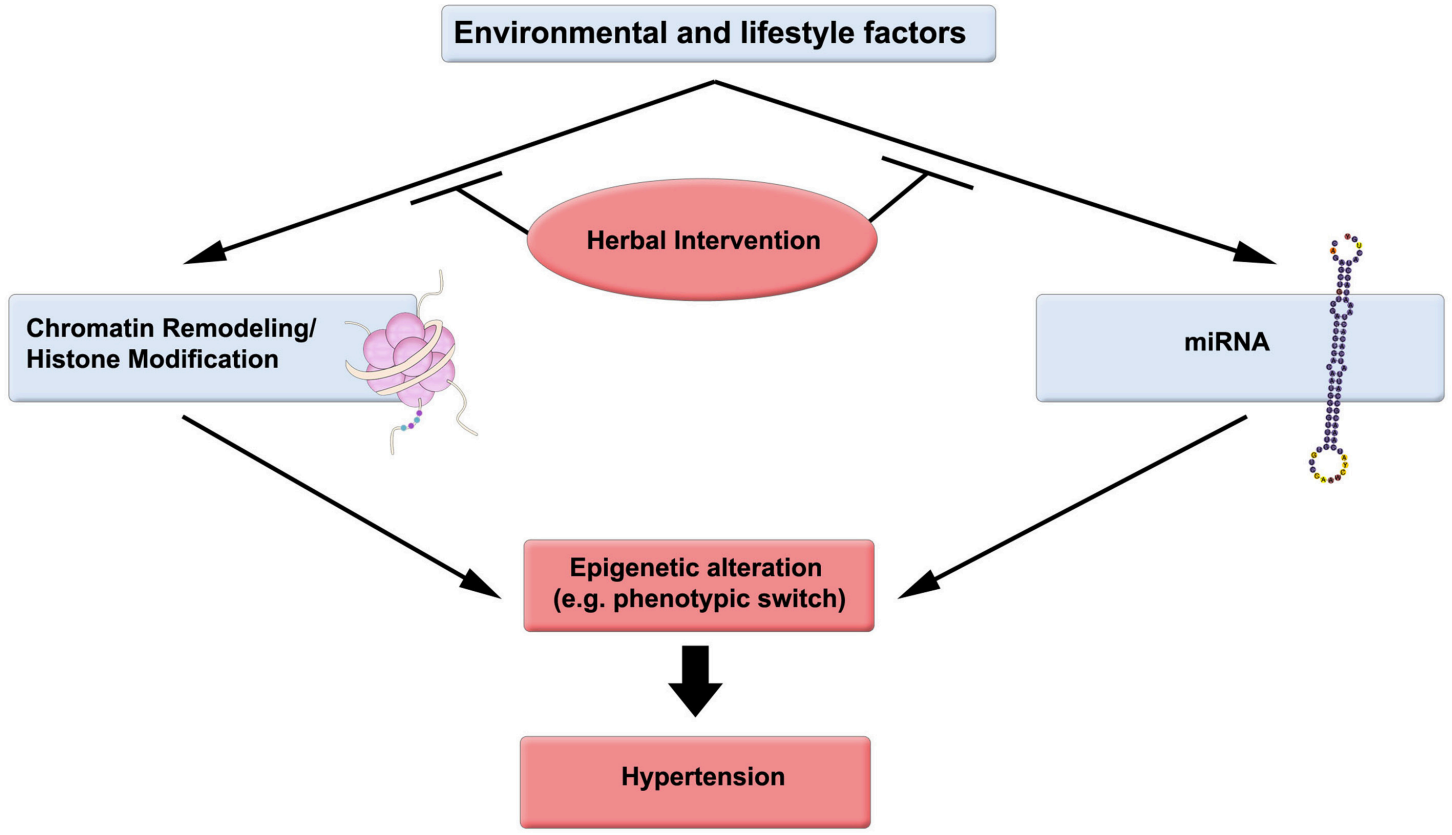
**Epigenetic-driven hypertension could be ameliorated by consumption of herbs and other factors**. Environmental and lifestyle factors such as diet (high fat and carbohydrate, low protein; minimal fruit, herb, and vegetable consumption), and Physical Activity (Lack of exercise—sedentary lifestyle) significantly affect DNA methylation, chromatin remodeling, and miRNA regulation. Herbal intervention can favorably modulate these epigenetic modifiers.

Epigenetic changes can be reversed chemically, and are receiving considerable attention by the scientific community and pharmaceutical industry. Pertinently, herbs and herb-derived molecules are at the forefront of research for their potential application in modulating the histone structure (Figure 1).

Recently, a comprehensive survey of Traditional Chinese Medicinals (TCMs), of which 95% are plants, was conducted. The goal of this study was to determine the potential epigenetic effects of these medicinals. Nearly 36% of these TCMs appeared to interact with human enzymes possessing histone-modifying capacities (Hsieh et al., 2013). Amongst this subset of therapeutic agents, 56% appeared to promote histone condensation (Hsieh et al., 2013), a mechanism that has significant implications in the pathophysiology of many diseases, including CVDs.

Methylation can be inhibited by Hesperidin (citrus) and Lycopene (tomatoes). Furthermore, catechins (present in tea), curcumin (in turmeric), and coumaric acid (in cinnamon) are known inhibitors of acetylation and methylation; and constituents of garlic (allyl sulfides) can also inhibit histone deacetylases (Kirk et al., 2008; Morimoto et al., 2008; Reuter et al., 2011; Shankar et al., 2013). A polyherbal mixture was recently shown to regulate class I and II histone deacetylases (Huang et al., 2014). Tanshinone 1, isolated from *Salvia miltiorrhiza* has been shown to repress acetylation of histone H3 (Gong et al., 2012). In addition, PPARγ, which is well-known for its role in cardiovascular physiology including the regulation of BP, is epigenetically regulated by rosmarinic acid (Yang et al., 2012).

As a note of caution, one drawback of these plant-derived bioactive components is their poor bioavailability; however, with structural modifications of the different parent molecules and/or improvement in delivery to identified locations will prove to be therapeutically beneficial. Clearly, further *in vivo* investigations are required to realize the full potential of these epigenomic modifiers.

### Micro RNAs (miRNAs)

Inclusion of non-coding RNAs (including miRNAs) in epigenomic machinery is a controversial subject (Choudhuri, 2011). However, both in health and disease states, non-coding RNAs play a major part in the regulation of epigenome, particularly targeting the associated enzymes at the posttranscriptional level (Iorio et al., 2010). MiRNAs, short-chain (~ 22) ribonucleotides, have received most of the attention compared to other members of the non-coding RNA group. Several hundreds have been identified, and increasing evidence implicates miRNAs in the development of hypertension (Marques et al., 2015). In this context, alternate expression of these molecular entities has been reported in hypertensive subjects, the expression of miR-27a, miR-150, and miR-192 was suppressed, whereas miR-92a, miR-130a, and miR-195 were upregulated in hypertensive patients (Marques et al., 2015). Moreover, blood concentrations of microRNAs (miR17, miR126, miR145, miR222) are associated with cardiovascular derangements, such as downregulation of eNOS that would lead to an increase in arterial tone and hence elevated BP (Baccarelli and Ghosh, 2012).

Interestingly, recent evidence shows that herbs and their components may play an important role in miRNA-related health benefits and pathologies (Stefanska et al., 2012; Wu et al., 2015). For example, grape extract has recently been shown to possess a beneficial immunomodulatory effect in hypertensives by virtue of its ability to downregulate miRNAs implicated in inflammation (Tomé-Carneiro et al., 2013).

## Limitations

Despite the multi-beneficial effects of consuming herbs in managing and treating several diseases, including hypertension, herbal medicine is not without many limitations. These include:

- Lack of sufficient quality control due to the absence of governmental or other health-related bodies overseeing the production and other relevant issues pertaining to herbal medicine, practice, and products.
- Undesired side effects which may not always be very obvious or immediate.
- Allergy to some components of the herb (or the herbal product).
- Seasonal variations in the content of the herbal plant parts (both aerial: bark, leaves, flowers, fruits seeds or stems, and underground: rhizomes or roots) and thus the potential quantitative changes in composition and/or ratios of bioactive ingredients.
- Drug interactions with other medicines.
- Adulteration of medications, where herbal prescriptions could be mixed with synthetic drugs like pain-killers or anti-inflammatory corticosteroids.
- Long time needed for an effect to be observed in some cases, which consequently precludes the use of the herb/herbal compound when an immediate resolution to the medical problem is needed.
- Limitations in certain diseases, since herbal medicine cannot be used to heal a concussion, broken bone or a heart attack.
- Misclassification of the herb/plant which may sometimes lead to misidentification.
- Lack of enough information on labels of bottles of herbal products, such as expiry dates (and how this affects the function; related to point “a” above).
- Lack of awareness among patients, physicians and the average public about the use or abuse of herbs and herb-related products.
- Self-medication due to over-the-counter availability of many herbal products.
- Large number of similar herbs in respective pharmacopeias.

## Conclusion

In this review, we highlighted the antihypertensive therapeutic benefits of plants and herbs. Premise of our argument is for a consistent, favorable outcome with herbal treatment for hypertension. The salient features cohesive to this thesis have illuminated the following: the augmentation of NO secretion via increase in expression and/or activity of eNOS; abatement of ROS levels through suppression of oxidant enzymes and an equilibrium shift toward increased expression of anti-oxidative enzymes and related molecules; inhibition of ACE and dyslipidemia. Together, all these favorable herb-modulated parameters are directed toward normalization in vascular function, alterations in actions of transcription factors (NF-κB), down-regulation of pro-inflammatory pathways, and improvement in renal physio-pharmacology (diuretic process). The overarching property of the aforementioned herbs is the reduction/normalization of BP.

A large percentage of hypertensive patients are certainly treatable by appropriate adaptation of life-style changes, such as shift from a sedentary (couch potato) setting to a more active, exercise-orientated healthy lifestyle. In addition to above, ingestion/consumption of functionally bioactive nutrients, such as the aforementioned phenolic-rich and other botanical-derived pharmacological agents, would be beneficial (“tonics” for most diseases) to normal health. Therefore, such judicious modifications will not only ameliorate hypertension, but simultaneously diminish the onset of athero-thrombogenic diseases (coronary arterial disease, peripheral arterial, and cerebrovascular diseases; Saleh Al-Shehabi et al., 2015). Moreover, the burden on scarce national health budgets will decrease.

Finally, there is an urgent need for more rigorous, well-developed clinical trials to obtain concrete evidence of beneficial impact of herbs and plants on hypertension and disease-free living. However, this must not detract from the evidence garnered herein from different animal models and human studies on the therapeutic benefits of herbs and plants.

## Author contributions

All authors contributed to the writing of this mansucipt. AE conceived, designed, and revised the manuscript.

### Conflict of interest statement

The authors declare that the research was conducted in the absence of any commercial or financial relationships that could be construed as a potential conflict of interest.
